# Hemoptysis: Comparison of Diagnostic Accuracy of Multi Detector CT Scan and Bronchoscopy

**DOI:** 10.5539/gjhs.v7n3p373

**Published:** 2015-04-24

**Authors:** Mohammad Davoodi, Mohsen Kordi, Mohammad Momen Gharibvand, Maryam Haddadzadeh Shoushtari, Hamid Borsi, Mohammad Bahadoram

**Affiliations:** 1 Department of Radio logy, Golestan Hospital, Ahvaz Jundishapur University of Medical Sciences, Ahvaz, Iran; 2 Departement of Internal Medicine, Division of Pulmonology, Imam Khomeini Hospital, Ahvaz Jundishapur University of Medical Sciences, Ahvaz, Iran; 3 Medical Student Research Committee, Ahvaz Jundishapur University of Medical Sciences, Ahvaz, Iran

**Keywords:** Radiography, MDCT, Bronchoscopy, Hemoptysis

## Abstract

**Background:**

Hemoptysis is the expectorating of blood from the tracheobronchial tree or pulmonary parenchyma. There is conflicting information about usefulness of radiography, MDCT, and bronchoscopy for investigating site and cause of the bleeding in patients with hemoptysis. The present study attempted to evaluated efficacy of these methods for identifying hemoptysis’ cause and etiology on 40 patients with the disease.

**Methods:**

A total of 40 patients with Hemoptysis who were referred to Golestan and Emam Khomeini hospitals were evaluated. Complete history of symptoms, volume and duration of Hemoptysis and demographic information were documented. Radiography, MDCT, and bronchoscopy were performed on all patients in order to investigate the site and cause of the bleeding.

**Results:**

Results showed MDCT had higher efficacy in identifying bleeding site than radiography, while efficacy of radiography and bronchoscopy or efficacy of MDCT and bronchoscopy weren’t significantly different. In addition, sensitivity of MDCT (60%) for detecting cause of the bleeding was higher than that of radiography (25%) and bronchoscopy (32.5%).

**Conclusion:**

The present study suggests MDCT as a suitable method in screening patients with hemoptysis, because it managed to detect site and causes of bleeding more efficiently than other methods. Additionally, we concluded that MDCT is an appropriate technique for diagnosing malignancies that cause hemoptysis in patients.

## 1. Introduction

Hemoptysis is the spitting of blood from the tracheobronchial tree or pulmonary parenchyma. The most important causes of the hemoptysis include tuberculosis, bronchiectasis, pneumonia, chronic bronchitis, malignancy, and fungal infections (ACR). Source and etiology of hemoptysis in the majority of patients are diagnosed primarily, while cryptogenic hemoptysis accounts for 3-42.2% of episodes of hemoptysis (Herth, Ernst & Becker, 2001; Bruzzi et al, 2006). Patients with hemoptysis should be carefully examined through history, physical examination, and diagnostic imaging techniques. Diagnostic imaging analysis is started with plain radiography and followed with MDCT or direct visualization with bronchoscopy, if a diagnosis remains unclear (Mal, Thabut & Plantier, 2003; Haponik, 1997; Nemati, Moghimi, Markazi Moghaddam & Amini, 2006; Jacob & Pacher, 2005). Formerly, after physical examination, MDCT scan was performed only for patients with abnormal radiography findings, but nowadays it is clear that MDCT scan should be performed for all hemoptysis patients (Lederle, Nichol & Parenti, 1986; Saldias & Leiva, 1997), because some patients with normal plain radiography were finally diagnosed with malignancy or other dangerous conditions (Nemati et al, 2006). The use of high-resolution MDCT has become more prevalent especially in parenchyma diseases (Jacob & Pacher, 2005). Bronchoscopy can effectively diagnose the site of bleeding and detect active hemorrhages, but is unable to localize the bleeding site efficiently (Bruzzi et al, 2006; Hsiao et al., 2001). In other words, the ability to localize the bleeding site is similar in MDCT, bronchoscopy, and radiography (Hsiao et al., 2001). There is conflicting data about the use of bronchoscopy and MDCT scan for evaluating hemoptysis patients. So, the current study was performed for clarifying efficacy of MDCT, bronchoscopy, and radiography in detecting and managing patients with hemoptysis history. In other words, this study was conducted for assessment of the plain radiography, MDCT, and bronchoscopy usefulness for diagnosing etiology, localization, and site of the bleeding in patients with hemoptysis.

## 2. Methods

### 2.1 Study Population

The present study was performed on 40 patients with history of significant hemoptysis (>10 cc) referred to Golestan and Emam Khomeini hospitals, Ahvaz Jundishapur University of the Medical Sciences, Ahvaz, Iran. Patients included 22 males and 18 females with mean age of the 44 yearsold (range: 22-77). Mean of hemoptysis duration was 8.2 days.

### 2.2 Study Intervention

On initial examination, patients were carefully assessed through history and physical examination. Data on age, clinical signs, bleeding volume, hemoptysis duration, coughing, and dyspnea were also recorded. Then patients were evaluated using three diagnostic imaging approaches including plain radiography, MDCT, and bronchoscopy. Patients with definite etiology on initial evaluation were excluded from the study. Bronchoscopy was performed by a pulmonologist. MDCT assay was also carried out using a standard protocol without contrast material and assessed by an expert radiologist. We compared the efficacy of MDCT and bronchoscopy for detecting the bleeding site and etiology of the hemoptysis.

### 2.3 Statistical Analysis

Data were described by relative frequency or mean± SD using SPSS 16. Software (Chicago, USA). Chi-squared test at significance level 0.05 was used for comparing tests results. In addition, the sensitivity of all the techniques was determined using a standard formula described below. Final diagnosis was considered as a gold standard.

Sensitivity= TP/(TP+FN)

Where: TP: true positive, FN: false negative.

## 3. Results

The current study evaluated and compared efficacy of radiography, MDCT, and bronchoscopy for detecting source and etiology of the patients with hemoptysis. Coughing and dyspnea were observed in 5% and 47.5% of patients respectively. In addition, hemoptysis volume was high in 37.5% of patients.

Our results indicated that radiography detected site of the bleeding in 52.5% of patients while MDCT and bronchoscopy identified site of the bleeding in 92.5% and 70% of patients respectively (Table 1). Analysis indicated that efficacy of MDCT for identifying bleeding site was statistically higher than radiography (*P*=0.05), while efficacy of the radiography and bronchoscopy or efficacy of MDCT and bronchoscopy were not significantly different (*P*>0.05). Radiography, MDCT, and bronchoscopy detected etiology in 25%, 60%, and 32.5% of patients with hemoptysis respectively (Table 2). We defined this ability as sensitivity and considered final diagnosis as a gold standard. Sensitivity of the radiography, MDCT, and bronchoscopy were 25%, 60%, and 32.5% respectively, which indicated higher sensitivity of MDCT than other methods. Each method also identified definite causes of hemoptysis in patients. These results were summarized in table 3. Because sample size was not large enough, resulting reliable sensitivity or specificity for each method wasn’t possible. However, as shown in table 3, radiography detected 83% TB, 67% pneumonia, and 100% tumor and cardiac cases properly, while MDCT diagnosed 67% TB, 100% tumor, bronchiectasis, Idiopathic pulmonary fibrosis (IPF), pneumonia, and cardiac cases properly. Both of these methods weren’t able to distinguish acute bronchitis. Bronchoscopy detected 33% TB and pneumonia cases, 18% bronchiectasis, and 100% acute bronchitis cases (Table 3).

**Table 1:** Descriptive analysis of the studied methods for detecting site of the bleeding

		Frequency	Percent
Radiography	None-detected	19	47.5
	Detected	21	52.5
MDCT	None-detected	3	7.5
	Detected	37	92.5
Bronchoscopy	None-detected	12	30.0
	Detected	28	70.0

**Table 2:** Descriptive analysis of the studied methods for detecting cause of the bleeding

		N (%)
Radiography	None-Detected	30 (75.0)
	Detected	10 (25.0)
MDCT	None-Detected	16 (40.0)
	Detected	24 (60.0)
Bronchoscopy	None-Detected	27 (67.5)
	Detected	13 (32.5)

**Table 3:** Descriptive analysis of the three methods for detecting definite cause of the bleeding

	Radiography	MDCT	Bronchoscopy	Final diagnosis
	N (%)	N (%)	N (%)	N (%)
Unknown	30(75.0)	16(40.0)	28(70.0)	8(20)
TB	5 (12.5)	4(10.0)	2(5.0)	6(15)
Bronchiectasis	-	12 (30.0)	2(5.0)	11(27.5)
Tumor	2 (5.0)	2(5.0)	-	2(0.5)
IPF	-	2(5.0)	-	2(0.5)
Pneumonia	2 (5.0)	3(7.5)	1(2.5)	3(0.75)
Cardiac	1 (2.5)	1(2.5)	-	1(0.25)
Acute Bronchitis	-	-	7 (17.5)	7(17.5)
Total	40(100)	40(100)	40(100)	40(100)

## 4. Discussion

We compared capacity of radiography, MDCT, and bronchoscopy to determine site and cause of patients with hemoptysis. Our results indicated that MDCT could detect source of the bleeding more efficiently than radiography. Although, radiography yielded more accurate results compared to other methods in diagnosing lesions resulting from TB. Revel et al (2002) showed site of the bleeding in 70%, 73%, and 45% of patients who underwent MDCT, bronchoscopy, and plain radiography, respectively. In accord with our study, Revel et al (2002) reported higher efficacy of MDCT than radiography and comparable ability of MDCT and bronchoscopy for identifying source of the hemoptysis (Revel et al., 2002). Nemati et al (2006) reported that bronchoscopy and MDCT detected lesion site in 22.5% and 27.5% of patients with hemoptysis (Nemati et al., 2006). However, Millar et al (1992) reported that MDCT is of value in the examination of patients with hemoptysis (Millar, Boothroyd, Edwards, Hetzel, 1992). Our results and previous investigations (Revel et al., 2002; Naidich, Funt, Ettenger & Arranda, 1990) concluded that MDCT could substitute bronchoscopy as the first-line technique for evaluating patients with hemoptysis.

Our finding showed radiography, MDCT, and bronchoscopy detected cause of hemoptysis in 25%, 60%, and 32.5% of patients respectively. Previous study indicated higher efficiency of MDCT than bronchoscopy for detecting the cause of bleeding (77% vs 8%, respectively) (Revel et al., 2002). Our study similarly showed higher sensitivity of MDCT than other methods. In addition, MDCT detected 100% tumor, IPF, pneumonia, and cardiac cases properly. In our study, MDCT and radiography precision in diagnosing tumoral lesions were found to be equal. This could be due to low sample volume in our study and MDCT’s higher precision in diagnosing pulmonary tumoral lesions and also radiography restrictions in diagnosing hypodiaphragm and posterior heart lesions. MDCT proved to be the most precise method in determining bronchiectasis lesions and IPF compared to other methods in our study. Set et al (1993) demonstrated that MDCT scan could identify all tumor samples, while bronchoscopy failed to detect two tumor samples (Set et al., 1993). Moreover, Haponik et al. (1987) confirmed that MDCT might have a complementary role in patients with risk factors for malignancy (Haponik, Britt, Smith & Bleecker, 1987). In a study by Thirumaran et al (2009) it was showed that MDCT detected 96% of patients with malignancy (Thirumaran, Sundar, Sutcliffe & Currie, 2009). As shown in results, similar findings were obtained by our study, because MDCT could detect all tumor samples while no tumor samples were identified by bronchoscopy. Furthermore, MDCT found all bronchiectasis samples while bronchoscopy found 18% of them. According to our study, bronchoscopy does not seem to be very helpful due to peripheral involvement in parenchymal lesions such as IPF, pneumonia and tumoral lesions. The site of IPF involvements is largely sub plural. Moreover, some pulmonary metastases and peripheral tumoral lesions in lungs may not be accessible to the bronchoscope due to not being endobrochial. In pneumonic patients, pus may not be found in bronchoscopy at all. But if seen, it could be the result of bronchiectasis, pneumonia, abscess, TB, etc. However bronchoscopy doesn’t help in determining its etiology. Bronchoscopy is an invasive procedure. Given the fact that MDCT is more accessible, less invasive and less expensive, it seems to be a better option in emergency circumstances.

## 5. Conclusion

In brief, our study supported the use of MDCT in screening patients presenting with hemoptysis, because it detected the site of bleeding more efficiently and had higher sensitivity in detecting the cause of hemoptysis. Moreover, our results confirmed MDCT as a suitable method for managing patients with hemoptysis resulting from malignancy.
